# Effects of Ordered Grasping Movement on Brain Function in the Performance Virtual Reality Task: A Near-Infrared Spectroscopy Study

**DOI:** 10.3389/fnhum.2022.798416

**Published:** 2022-03-31

**Authors:** Xiangyang Li, Jiahui Yin, Huiyuan Li, Gongcheng Xu, Congcong Huo, Hui Xie, Wenhao Li, Jizhong Liu, Zengyong Li

**Affiliations:** ^1^Nanchang Key Laboratory of Medical and Technology Research, Nanchang University, Nanchang, China; ^2^Beijing Key Laboratory of Rehabilitation Technical Aids for Old-Age Disability, National Research Center for Rehabilitation Technical Aids, Beijing, China; ^3^Key Laboratory for Biomechanics and Mechanobiology of Ministry of Education, School of Biological Science and Medical Engineering, Beihang University, Beijing, China; ^4^Key Laboratory of Neuro-Functional Information and Rehabilitation Engineering of the Ministry of Civil Affairs, Beijing, China

**Keywords:** virtual reality, hand functional practice, functional near-infrared spectroscopy, kinematic characteristics, functional connectivity

## Abstract

**Objective:**

Virtual reality (VR) grasping exercise training helps patients participate actively in their recovery and is a critical approach to the rehabilitation of hand dysfunction. This study aimed to explore the effects of active participation and VR grasping on brain function combined with the kinematic information obtained during VR exercises.

**Methods:**

The cerebral oxygenation signals of the prefrontal cortex (LPFC/RPFC), the motor cortex (LMC/RMC), and the occipital cortex (LOC/ROC) were measured by functional near-infrared spectroscopy (fNIRS) in 18 young people during the resting state, grasping movements, and VR grasping movements. The EPPlus plug-in was used to collect the hand motion data during simulated interactive grasping. The wavelet amplitude (WA) of each cerebral cortex and the wavelet phase coherence (WPCO) of each pair of channels were calculated by wavelet analysis. The total difference in acceleration difference of the hand in the VR grasping movements was calculated to acquire kinematic characteristics (KCs). The cortical activation and brain functional connectivity (FC) of each brain region were compared and analyzed, and a significant correlation was found between VR grasping movements and brain region activation.

**Results:**

Compared with the resting state, the WA values of LPFC, RPFC, LMC, RMC, and ROC increased during the grasping movements and the VR grasping movements, these changes were significant in LPFC (*p* = 0.0093) and LMC (*p* = 0.0007). The WA values of LMC (*p* = 0.0057) in the VR grasping movements were significantly higher than those in the grasping movements. The WPCO of the cerebral cortex increased during grasping exercise compared with the resting state. Nevertheless, the number of significant functional connections during VR grasping decreased significantly, and only the WPCO strength between the LPFC and LMC was enhanced. The increased WA of the LPFC, RPFC, LMC, and RMC during VR grasping movements compared with the resting state showed a significant negative correlation with KCs (*p* < 0.001).

**Conclusion:**

The VR grasping movements can improve the activation and FC intensity of the ipsilateral brain region, inhibit the FC of the contralateral brain region, and reduce the quantity of brain resources allocated to the task. Thus, ordered grasping exercises can enhance active participation in rehabilitation and help to improve brain function.

## Introduction

Stroke has gradually become the second leading cause of death worldwide among people over 60 years old in the world, and it is also the leading cause of disability (Murray et al., 2012; Lozano et al., 2012, 2013; Mozaffarian et al., 2015). Most stroke patients suffer limb motor dysfunction, and 50% of the survivors still have upper extremity dysfunction 2–4 years after stroke, which not only seriously affects their daily life, social participation, functional roles, and the independence in leisure activities, but also imposes a heavy burden on the family and society (Faria-Fortini et al., 2011; Morris et al., 2013). Therefore, various interventions are emerging to improve the upper extremity function: among these treatments, repetitive high-intensity repetitive task-specific training in particular seems to bring tremendous benefits (Jutai and Teasell, 2003).

Neuroplasticity is a widely studied concept and provides a theoretical basis for improving the effectiveness of rehabilitation in stroke patients (Dimyan and Cohen, 2011). Cerebral cortex reorganization plays an important role in neuroplasticity: therefore, a sharp decrease in the activity of hemiplegic limbs after stroke may lead to a decrease in neuroplasticity (Johansson, 2011). However, limited medical resources and high medical costs (Rosati, 2010) obstruct patients from obtaining proper medical treatments. Worse, the monotony and high intensity of repetitive training tasks may cause the patients to experience burnout and ultimately decrease the effectiveness of treatment (Burdea, 2003). Therefore, virtual reality (VR) may be a useful tool for rehabilitation exercises. First, VR can reduce the amount of physician labor required for the rehabilitation process; additionally, by aligning with users’ interests, VR can keep the patients in a positive mood, improve their initiative and enthusiasm in rehabilitation training, and improve the effectiveness of rehabilitation (MacLellan et al., 2020); finally, VR can also collect objective data from rehabilitation training to help doctors understand their patients’ rehabilitation status of patients and carry out targeted training.

Repeated grasping exercise training is very beneficial to the rehabilitation of hand function in patients with cerebral apoplexy (Park et al., 2020). However, simple repetitive exercise can easily cause fatigue during training, which can reduce patients’ initiative to participate in training, affect the efficiency of rehabilitation, and even cause patients to miss the optimal time window for rehabilitation. The integration of VR technology to achieve VR grasp movements training can improve patients’ active participation in training, help patients improve active participation, and improve the efficiency of rehabilitation (MacLellan et al., 2020).

Functional near-infrared spectroscopy (fNIRS), a new type of functional neuroimaging technology, is a non-invasive technique for monitoring and imaging cerebral hemodynamic function without exposing the subject to ionizing radiation; this technique is often used to study human brain function and various pathologies (Regan et al., 2008). Because the absorption rate of hemoglobin to light above wavelength 650 nm is low, and near infrared light (65–1,000 nm) can pass through the scalp and skull of human body and spread in human tissues in the main way of scattering, it is possible to detect the concentration changes of oxygenated hemoglobin, deoxyhemoglobin, and total hemoglobin in cerebral cortex by spectral method (Boas et al., 2014). fNIRS has the advantages of relatively low cost, simple setup, and few physical/environmental restrictions or contraindications (Scholkmann et al., 2014). Therefore, it can be easily be used to detect hemodynamic fluctuations for structural and functional analysis of the brain in a clinical environment. Functional connectivity (FC) is defined as the strong temporal correlation between two raw sequences of brain activity signals in the low-frequency band (Cordes et al., 2001). In a certain fNIRS signal time, the period number of the high frequency component is larger than that of the low frequency component, and compared with the high frequency component, the phase difference of the low frequency component tends to be constant (Bernjak et al., 2012). Thus, FC reflects the critical relationship between the cortical regions of the brain and helps reveal the inherent characteristics of the brain network (Biswal et al., 1995; Fox and Raichle, 2007).

Rehabilitation exercise training can cause complex changes in the brain, including changes in cortical excitability and cortical FC (Cicinelli et al., 1997; Traversa et al., 1997; Caliandro et al., 2017). However, the neuroplasticity changes in the cerebral cortex during motor rehabilitation with VR grasping exercises have not been fully elucidated. This study aims to explore the effects of VR grasping exercises on human brain function via fNIRS. We hypothesized that VR grasping exercises could induce cortical activation and FC changes in the trainee.

## Materials and Methods

### Subjects

A total of 18 young subjects (8 females and 10 males) were enrolled in this experiment. All subjects were 25 ± 5 years old and able to initiate grasping movements. All subjects were strongly right-handed according to the Edinburgh handedness inventory (Oldfield, 1971). Subjects with any of the following characteristics were excluded: congenital malformations in any part of the body, infectious diseases, critical illnesses, or poor compliance. We collected the subjects’ age, body mass index (BMI), and handedness. The experimental procedure was approved by the Human Ethics Committee of the National Research Center for Rehabilitation Technical Aids and was in accordance with the ethical standards specified by the Helsinki Declaration of 1975, Declaration of Helsinki as revised in 2008. The contract number is “ChiCTR210005148.” All subjects signed informed consent forms before inclusion. Statistical information on the participants’ characteristics is shown in Table 1.

**TABLE 1:** Basic information of the subjects.

Parameters	Means (*SD*)
Age (years)	25.2 (1.9)
Body mass index (BMI)	21.21 (2.6)
Number of right-handed people	18 (0)

*The data shown in the table is presented in the form of the mean (standard deviation).*

### Virtual Reality Game Design

After the upper motor system of the central nervous system is damaged in stroke patients, the flexion and extension speed of the affected hand become significantly slower than those of the unaffected hand, the range of motion is narrowed, and coordination between joints is impaired (Rand et al., 2000; Cirstea et al., 2003). In view of this symptom, we designed a VR rehabilitation training task for stroke patients in the form of a game, not only to improve their initiative and enthusiasm for rehabilitation training but also to better promote the recovery of their hand function.

**FIGURE 1:**
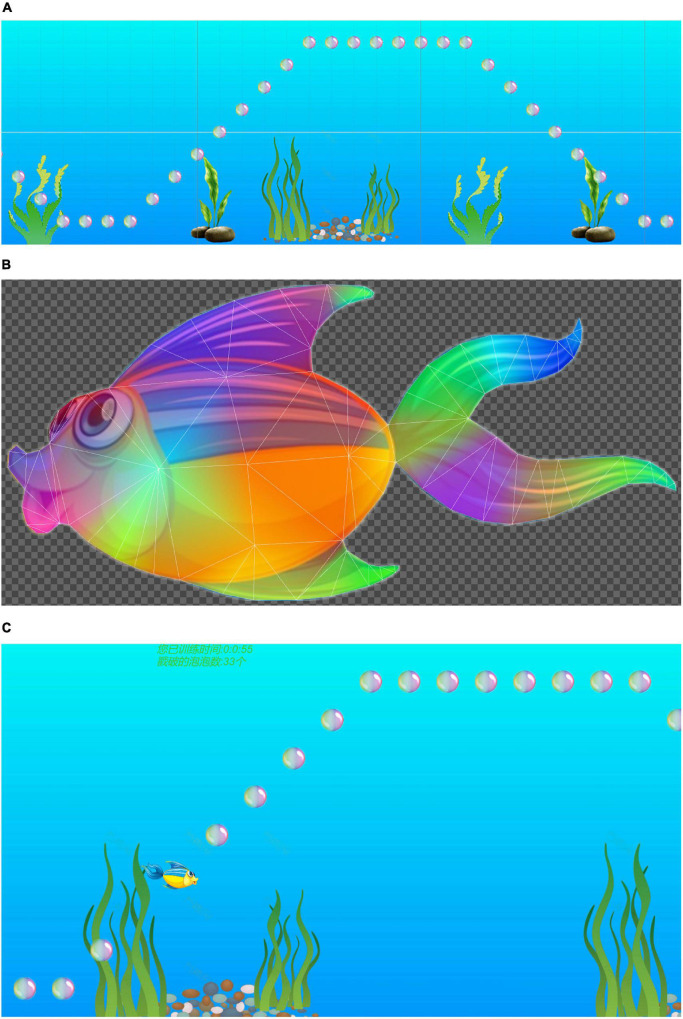
**(A)** The frequency of the arrangement of bubbles, and the arrangement of bubbles is continuously repeated. **(B)** In order to increase the enjoyment of the game and improve the enthusiasm of the user, the animation effect of the fish is realized. **(C)** VR scene includes scores, path, and training time records.

Unity3D was used to design the VR scenes required in the experiment; the advantages of this software included its image rendering engine, its physical effects engine, and the availability of related art assets. In post stroke hand function rehabilitation training, intense repetitive movements can achieve a satisfactory therapeutic effect (Burdea, 2003), and maintaining the maximum challenge level for 7–8 s produced the best effect during grasp training exercises. Therefore, the trajectory formed by the arrangement of bubbles was maintained at the highest challenge level and a sufficiently long distance. In addition, the same arrangement of bubbles was presented repeatedly, and the subsequent tracks will repeat this paragraph all the time, which is also to reduce the test variables (Figure 1A). And the participants were to reach along this trajectory each time. The participants succeeded in repeating this sequence of grasping motions continuously during the experiment. To increase the user’s enjoyment of the game and improve enthusiasm for the task, the animation component of Unity3D was used to animate a fish with a naturalistic swimming motion (Figure 1B). Scores and training time records (Figure 1C) were added to the game, giving the user an easy way to track in-game progress and duration of play, which was intended to inspire a sense of achievement and stimulate enthusiasm for the task.

**FIGURE 2:**
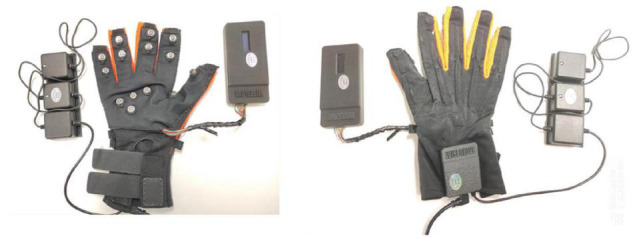
Wiseglove14 data glove.

Through digital-to-analog conversion, the bending sensor signal of the Wiseglove14 data glove (Figure 2) controlled the movements of the fish in the training simulation. In the experiment, the participants used a right-handed Wiseglove14 data glove to interface with the VR game as it ran on a PC. The gripping motion of the hand was used as an interactive signal to control the movements of the fish in the game; the participant’s task was to use the fish to collect as many bubbles as possible (Figure 1C). Furthermore, owing the fixed trajectory marked out in the training scene, the participants could perform grasping movements in an orderly manner in a semi-immersive VR environment.

As the participants performed the VR task, the EPPlus plug-in was used in Unity3D to collect hand motion data during the VR grasping motions, and a file in.xlsx format was created to collect the acquired data, including the elapsed gameplay time, the bending angle of the fingers, the real-time coordinates of the fish, and the coordinates of the user’s hand along the planned trajectory at each time.

### Experimental Procedure

**FIGURE 3:**
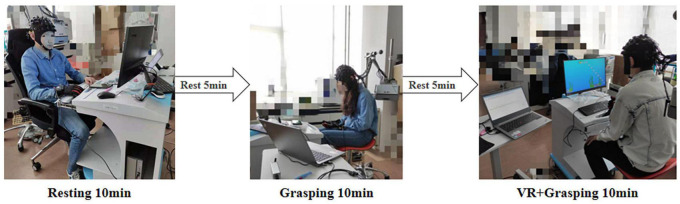
Experimental process and scenario. First, the subjects rest for 10 min, then rest for 5 min, then grasping movements for 10 min, then rest for 5 min, and finally VR grasping movements for 10 min.

This experiment was separated into three states: the resting state, grasping movements, and VR grasping movements. Each state lasted for 10 min, with intervals of 5 min between states to prevent fatigue. Figure 3 illustrates the procedure for the experiment.

Before starting the experiment, participants were familiarized with the VR game through at least 10 min of practice so that their average bubble collection rate at each distance exceeded 95% (Buetefisch et al., 2014).

In the resting state, the participants sat still for 10 min in a comfortable position with their eyes closed while wearing the sensor glove. After a 5-min break, the participants executed untargeted non-VR grasping exercises for 10 min while wearing the sensor glove. After another 5-min rest, the participants wore the sensor glove while performing VR grasping exercises for 10 min. In this experimental task, bubbles were presented repeatedly in the same arrangement within the VR scene, and participants guided the animated fish through the scene to pierce as many bubbles as possible. In other words, participants performed a series of ordered grasping movements.

In the experiment, with the guidance of the VR scene, the grip movements is a grip with fixed frequency, which we call orderly grasping exercises, otherwise it is disorderly grasping exercises. In order to control the test variables, we didn’t add any other sounds in each experiment. Throughout the experiment, fNIRS signals were collected continuously.

### Functional Near-Infrared Spectroscopy

fNIRS was performed using a multichannel tissue oxygenation monitor (NirSmart of Danyang Huicuang Medical Equipment Co., Ltd., Danyang, China). After fixing the near-infrared hat to the subjects, used the tool to pull the subjects’ hair aside to expose the scalp, and ensured the probes close to the scalp. The position of the brain region used in the measurement was 10/10 electrodes (Oostenveld and Praamstra, 2001).

In selecting the brain regions of interest, it was necessary to take into account the areas of the cerebral cortex responsible for grip movements and performance of the VR task. The prefrontal cortex (PFC) is generally considered to be the center of cognitive function in human beings (Koechlin et al., 1999; Mandrick et al., 2013), and participates in the executive processes, including working memory, allocation of attention resources, and processing of information related to planning (MacLeod et al., 1998). Therefore, the PFC is a suitable region in which to study the changes in brain activity during VR training. The motor cortex (MC) participates in sensing the posture and movement of the human body and controls the movement of the contralateral limbs, which plays an important role in sensation and motor control (Peterka, 2002). The occipital cortex (OC) is mainly involved in visual information processing and is also associated with functions such as memory and motion perception (Astafiev et al., 2004).

**FIGURE 4:**
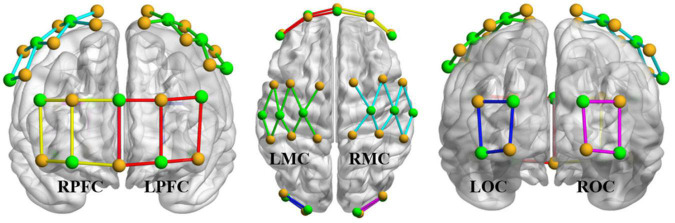
Light source (yellow), probe (green), and channel layout. The yellow dot represents the light source, the green dot represents the detector, the LPFC channel is marked with the red line segment, the RPFC channel is marked with the yellow line segment, the LMC channel is marked with the green line segment, the RMC channel is marked with the cyan line segment, the LOC channel is marked with the blue line segment, and the ROC channel is marked with the pink line segment.

Therefore, in the experiment, visual markers and templates were placed in six cortical regions: left PFC (LPFC), right PFC (RPFC), left MC (LMC), right MC (RMC), left OC (LOC), and right OC (ROC). The distance between the visual markers was 30 mm. As shown in Figure 4, a 41-channel fNIRS design was used in the experiment. In the picture, the yellow dot represents the light source, the green dot represents the detector, the LPFC channel is marked with the red line segment, the RPFC channel is marked with the yellow line segment, the LMC channel is marked with the green line segment, the RMC channel is marked with the cyan line segment, the LOC channel is marked with the blue line segment, and the ROC channel is marked with the pink line segment. Signals with low signal-to-noise ratios were excluded. The sampling rate was 10 Hz.

Subjects were to keep their heads still during the experiment. During the VR grasping task, the Excel file of one subject’s hand motion data could not be saved; thus, only 17 participants yielded valid Excel files of hand motion data in this state.

**FIGURE 5:**
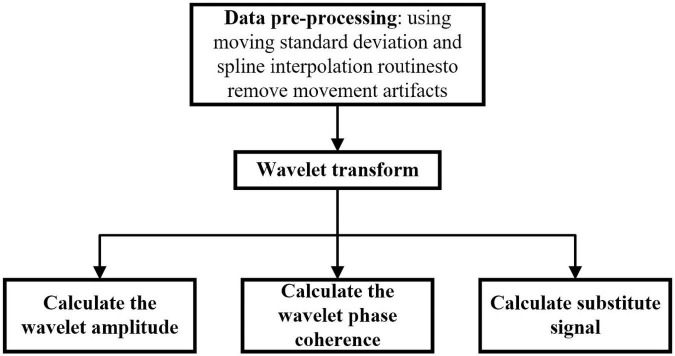
Data processing process. First, pre-process the data of fNIRS, then calculate the wavelet transform, and finally calculate the wavelet amplitude, the wavelet phase coherence and substitute signal.

Figure 5 shows the process of the data processing. First, pre-process the data of fNIRS, then calculate the wavelet transform, and finally calculate the wavelet amplitude, the wavelet phase coherence and substitute signal. The specific processing methods are as follows.

### Data Pre-processing and Analysis

Previous works have demonstrated that ΔHbO_2_ signals present a better signal-to-noise ratio than ΔHHb signals. Additionally, ΔHbO_2_ variables in the cerebral cortex are sensitive to information processing and change significantly during attention-related tasks, and the combined analysis of ΔHbO_2_ and HHb variables is, in some cases, only a slight improvement over ΔHbO_2_ analysis alone (Kameyama et al., 2006; Derosiere et al., 2014). Therefore, in this study, only ΔHbO_2_ signals were used for the wavelet amplitude (WA) and the wavelet phase coherence (WPCO) analysis.

In this study, 0.01–0.08 Hz was selected as the fNIRS signal band of interest. In order to perform covariance analysis, resting state brain network indicators are required as covariates. The low-frequency fluctuations (0.01–0.08 Hz) of fNIRS signals in resting state have been considered to be physiologically important and may reflect spontaneous neural activity (Tan et al., 2015). Our previous studies have reported the network connections related to endothelial cell metabolic activity, neural activity and myogenic activity in this band in healthy people and stroke patients. In addition, this band of interest avoids the interference of mayer waves (∼0.1 Hz), which may reduce the accuracy of fNIRS estimation and analysis. In order to obtain a better fNIRS low-frequency fluctuations (0.01–0.08 Hz) signal, we used the moving average method and the three-order Butterworth filter to obtain the filtered signal with high signal-to-noise ratio (SNR). The time window for the moving average filter was 3 s. Motion artifacts in visual recognition channels were eliminated using moving standard deviation and spline interpolation programs (Uchiyama et al., 2003). Ultimately, the preprocessing yielded the cerebral blood oxygen signal in the range of 0.01–0.08 Hz with an acceptable signal-to-noise ratio.

### Wavelet Transform and Calculate Wavelet Amplitude

The wavelet transform is a complex transform of time series from the time domain to the time-frequency domain; the appropriate time and frequency resolution are achieved by using adjustable filter band length. In this study, the Morlet complex wavelet was selected as the wavelet basis function for blood oxygen signal oscillations to extract multiple periods of the time series and extract the trend on different time scales (Addison et al., 2006).

The calculation formula of wavelet transform is as follows:


(1) $$X\,\left(s,t\right)=\int_{-\infty }^{+\infty }H_{s,t}\,\left(u\right)\,x\,\left(u\right)\,du$$

*Note: X(s,t) is the result of wavelet transform, x(u) is the original signal, and *H*_*s*,*t*_(*u*) is the wavelet basis function, the definition of *H*_*s*,*t*_(*u*) as the formula 2.*

(2) $$H_{s,t}(u=\frac{1}{\sqrt{s}}H\left(\frac{u-t}{s}\right)$$

*Note: s is the scale sequence of wavelet transform, t is the time parameter.*

Through averaging the results of wavelet transform in time domain, we can get the wavelet amplitude (WA) of each ΔHbO_2_ signals. Among the detected fNIRS signals, wavelet amplitude (WA) is used to characterize the intensity of activity in specific cortical regions. When a cortical region is activated, the corresponding WA value will increase due to increased cerebral blood flow into that region; therefore, the increase in the WA value represents the specific activation of that cortical region induced by the task.

Consider the fNIRS signal of a subject performing a task. After data preprocessing, the wavelet transform results of ΔHbO_2_ signals from 41 channels could be calculated by the wavelet transform analysis method. After integral averaging of the results in the time domain, the WA value in the frequency domain was obtained. Then, the WA values of the 41 channels were integrated using the trapezoidal rule in the frequency range of 0.01–0.08 Hz, and the area as divided by the width of the frequency interval, yielding the mean WA values of these 41 channels in the frequency range of 0.01–0.08 Hz. Finally, the mean WA values of the channels contained in the six cortical regions of interest (LPFC, RPFC, LMC, RMC, LOC, and ROC) were added and then divided by the number of channels in the brain region. In this manner, the WA mean values of the six cortical regions of the participants in the frequency domain of 0.01–0.08 Hz were obtained for each task.

### Calculate Wavelet Phase Coherence and Analysis Functional Connectivity

After the wavelet transform, by subtracting the instantaneous phase, we can obtain the instantaneous phase difference between each pair of channels ΔHbO_2_ signals: Δφ(f,*t*_*n*_). Then through average cos(Δφ(f,*t*_*n*_)) (Formula 3) and cos(Δφ(f,*t*_*n*_)) (Formula 4) in time domain, we get [cos(Δφ(f,*t*_*n*_))] and[sin(Δφ(f,*t*_*n*_)].


(3) $$\left[\text{cos}\,\left(\Delta \,\varphi \,\left(f,t_{n}\right)\right)\right]=\frac{1}{\text{M}}\,\sum_{m=1}^{M}\text{cos}\,\left(\Delta \,\varphi \,\left(f,t_{m}\right)\right)$$

(4) $$\left[\text{sin}\,\left(\Delta \,\varphi \,\left(f,t_{n}\right)\right)\right]=\frac{1}{\text{M}}\,\sum_{m=1}^{M}\text{s}\,i\,n\,\left(\Delta \,\varphi \,\left(f,t_{m}\right)\right)$$

*Note: m = 1,2,…,M, M is the number of data contained in the ΔHbO_2_ signal data sequence.*

Finally, we obtain WPCO through calculated formula 5.


(5) $$W\,\left(f\right)=\sqrt{{\left(\left[\sin\left(\Delta \,\varphi \,\left(f,t_{n}\right)\right)\right]\right)}^{2}+{\left(\left[\sin\left(\Delta \,\varphi \,\left(f,t_{n}\right)\right)\right]\right)}^{2}}$$

Take the fNIRS signal of a subject performing a task as an example. After data preprocessing, the WPCO value between each pair of channels is calculated by the wavelet phase coherence analysis method. And then, the WPCO value in the frequency domain of 0.08 Hz is integrated to obtain the mean value of WPCO between the two channels in the frequency domain of 0.01–0.08 Hz in this state (Xu et al., 2017). The mean WPCO between each pair of channels in each state can be calculated according to this calculation method.

### Calculate Substitute Signal

To intuitively compare the changes in FC in brain regions during different task states, FC diagrams can be drawn. The drawing method was as follows: first, using the above calculation method, the mean and standard deviation of the WPCO for 100 substitute signals between two channels were calculated in the frequency range of 0.01–0.08 Hz. In this task state, the mean WPCO between each pair of channels was compared with the mean WPCO of the substitute signal plus twice the standard deviation. If the mean value of WPCO in the task state is larger than the WPCO mean of the substitute signal plus the sum of twice the standard deviation, which suggests a FC between the two channels (Xu et al., 2017), the mean value of the WPCO was retained. Otherwise, FC was considered to be absent, and the mean value of WPCO returned to zero. The FC diagram was drawn accordingly.

### Virtual Reality Behavior Data Analysis and Statistical Analysis

When each VR grasping movements ended, the temporal task state, the locations of the fish (S), and the planned path (S’) were recorded and collected in an Excel file. Based on the data, the total distance difference (TDD), sum velocity difference (SVD), and total acceleration difference (TAD) were derived via Equations (1)–(3). TDD, SVD, and TAD were used as kinematic characteristics (KCs) of movements in the VR task.


(6) TDD=∑n=22500|ΔSn-ΔSn|′

(7) $$\text{SVD}=\sum_{\text{n}=2}^{2500}\left|\frac{\Delta S_{n}-\Delta S_{n}{}^{\prime }}{\Delta \,t_{n}}\right|$$

(8) $$\text{TAD}=\sum_{\text{n}=3}^{2500}\left|\frac{\frac{\Delta \,S_{n}}{\Delta \,t_{n}}-\frac{\Delta \,S_{n-1}}{\Delta \,t_{n-1}}}{\Delta \,t_{n}-\Delta \,t_{n-1}}-\frac{\frac{\Delta S_{n}{}^{\prime }}{\Delta \,t_{n}}-\frac{\Delta S_{n-1}{}^{\prime }}{\Delta \,t_{n-1}}}{\Delta \,t_{n}-\Delta \,t_{n-1}}\right|$$

*Note:*Δ*S*_*n*_=*s*_*n*_−*s*_*n*−1_, Δ*t*_*n*_=*t*_*n*_−*t*_*n*−1_,*n represents the valid data points collected during the VR grip movements, and 2,500 is the total number of valid data points.*

### Statistical Analysis

In the statistical analysis software SPSS 20.0, each subject’s data were subjected to a normality test (Kolmogorov-Smirnov test) and a test of homogeneity of variance (Levene’s test) to ensure that the data fulfilled the assumptions for a parametric test. The significant difference in WA value of each brain region and the significant change of WPCO value between each pair of channels, were calculated by one-way ANOVA. If *p* < 0.05, the difference is statistically significant. The Bonferroni *t*-test was used for the pair-wise comparisons. Since three pairs of variables (Resting state and Grasping movements, Resting state and VR grasping movements, Grasping movements and VR grasping movements) of WA and WPCO were considered, the α-value was adjusted to 0.05/3 = 0.0167 using the Bonferroni correction. Pearson correlation coefficient test was performed to compare the correlation and significance between WA with TDD, SVD and TAD in VR grasping movements.

## Results

### Significance Amplitude Differences

**FIGURE 6:**
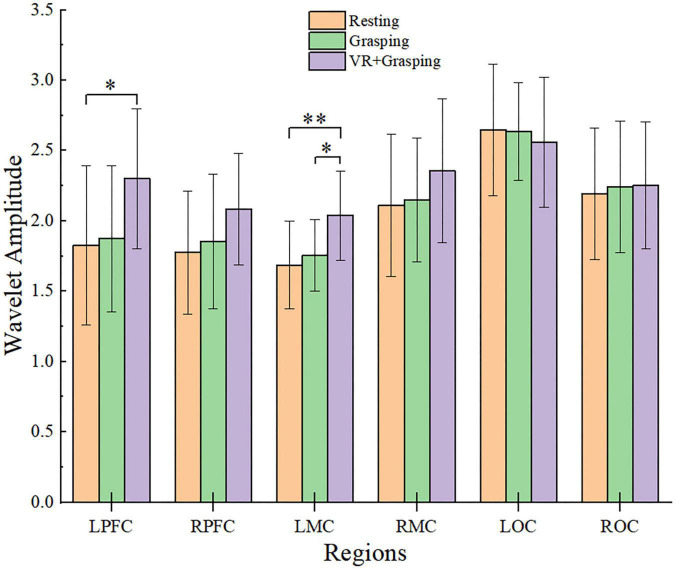
Amplitude changes of brain regions in different states and the comparison results, * were used to mark significant differences, among which *(*p* < 0.0167), ^**^(*p* < 0.001). The yellow column represents the resting state, the green column represents the grasping movements, and the purple column represents the VR grasping movements.

The WA values of the six cerebral regions were assessed and compared in three states, namely, the resting state, grasping movements, and VR grasping movements, as shown in Figure 6. It can be seen intuitively from the figure that the WA values of the LPFC, RPFC, LMC, RMC, and ROC all increased from resting to grasping to VR grasping. In particular, the WA values of the LPFC (*p* = 0.009) and LMC (*p* < 0.001) were significantly higher during VR grasping movements than in the resting state, and the WA values of the LMC (*p* = 0.006) in VR grasping movements were significantly higher than those in grasping movements.

### Results of Functional Connectivity Analysis

**FIGURE 7:**
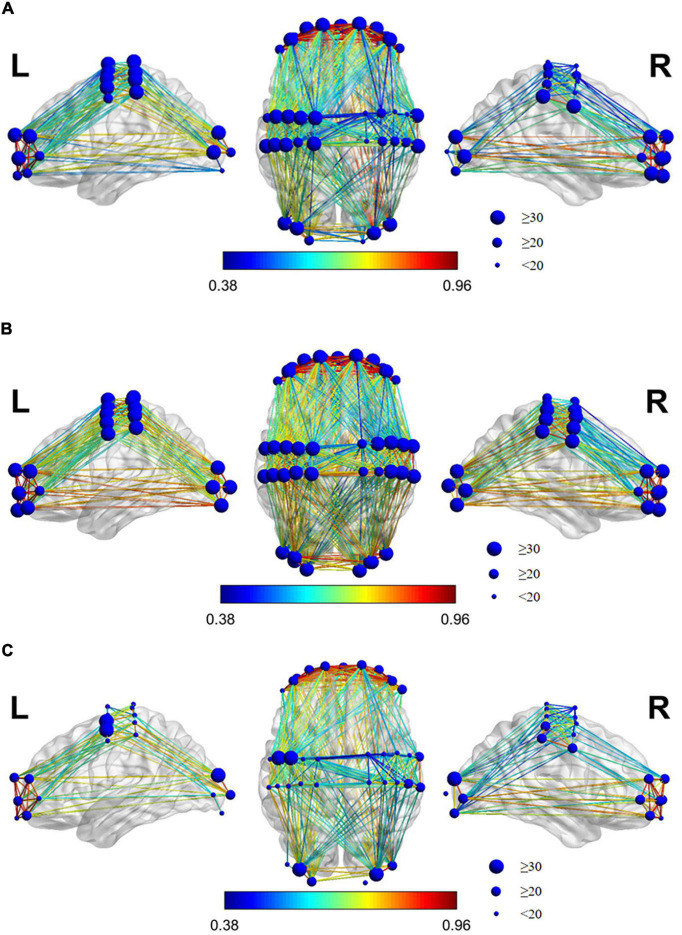
Brain FC map of the state of each task. **(A)** Resting, **(B)** Grasping, **(C)** VR Grasping. If there is a connection line between the two channels, it shows a FC between the two channels with a significant WPCO value. The redder the connection in the picture, the stronger the FC between the two channels, and the bluer the connection, which means the weaker the FC between the two channels. The size of the blue dot on the channel location reflects the number of connection between the channel and all other channels.

Figure 7 shows the brain’s FC in the three states of resting, grasping, and VR grasping. If there is a line connecting two channels, it represents significant FC between those channels, with a significant WPCO value. The redder the connecting line in the picture, the stronger the FC between the two channels; the bluer the line, the weaker the FC. The size of the blue dot at each channel location reflects the number of FC connections between that channel and all others.

As shown in the FC diagrams of each state, there was always marked, high-intensity FC between the LPFC and RPFC channels (WPCO > 0.8). The FC between the channels was significantly increased and intensified during grasping movements compared to the resting state. Additionally, the FC between the channels was significantly reduced and weakened during VR grasping movements compared with resting or non-VR grasping, and the FC between channels was concentrated between the LMC and LPFC, between the LMC and RPFC, and between the LMC and LOC.

The comparison of FC diagrams can qualitatively reveal the change in connectivity between different states, while the result of single-factor analysis of variance of WPCO values can quantitatively measure the significance of the change in FC.

**FIGURE 8:**
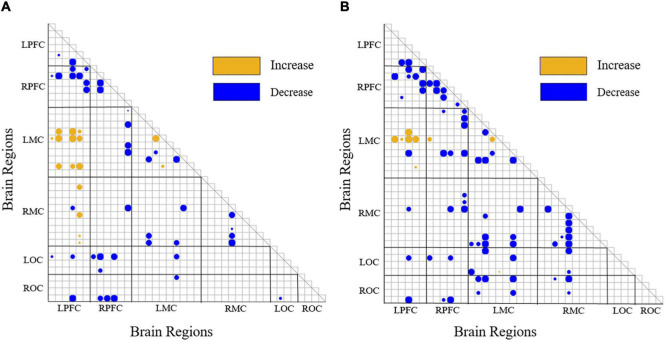
Significance matrix of WPCO value change. yellow color suggested that there was a significant increase of WPCO value between two states (*p* < 0.0167), blue color represented that there was a significant decrease of WPCO value between two states (*p* < 0.0167). **(A)** The significance matrix of the change of WPCO value between each pair of channels of VR grasping movements compared with that of resting state. **(B)** The significance matrix of the change of WPCO value between each pair of channels of VR grasping movements compared with that of grasping movements.

Figure 8 shows the significance matrix of the change of WPCO value between two channels in each state. Only if there exists a significant difference in the WPCO value of the two channels will the corresponding boxes be filled with circular color clocks. And the larger blocks suggest higher significance. In Figure 8, yellow color suggested that there was a significant increase of WPCO value between two states (*p* < 0.0167), blue color represented that there was a significant decrease of WPCO value between two states (*p* < 0.0167), and larger circle color block indicated that the significance was stronger.

Because there is no significant change in the WPCO value between all the two channels in the grasping motion relative to the resting state, the significance matrix diagram of the WPCO value change is not drawn. Figure 8A shows the significance matrix of the change of WPCO value between each pair of channels of VR grasping movements compared with that of resting state. The WPCO value between LPFC-LMC partial channels increases significantly (*p* < 0.0167). In addition, the WPCO value between a small portion of LPFC-RMC channels increases significantly (*p* < 0.0167), while the WPCO value between LPFC-RPFC, RPFC-LMC, LMC-RMC, RPFC-LOC, and RPFC-ROC parts decreases significantly (*p* < 0.0167). Figure 8B shows the significance matrix of the change of WPCO value between each pair of channels of VR grasping movements compared with that of grasping movements; the WPCO value between LPFC-LMC partial channels increases significantly (*p* < 0.0167). In addition, the WPCO value between LPFC-RMC channels does not increase significantly (*p* < 0.0167), and the WPCO values between LPFC-RPFC, RPFC-LMC, RPFC-RMC, LMC-RMC, LMC-LOC, and LMC-ROC partial channels decrease significantly. On the whole, except for LPFC-LMC, the FC between almost all channels in VR grasping movements group decreased significantly compared with grasping movements.

### Analysis Results of Virtual Reality Behavior Data

Table 2 shows a list of the Pearson correlation results. As can be seen from the table, in the state of VR grasping movements, the WA of LPFC, RPFC, LMC, and RMC is negatively correlated with TDD, SVD, and TAD (*R* < 0), on the contrary, the WA of LOC and ROC is positively correlated with TDD, SVD and TAD (*R* > 0). It was observed that there was a significant negative correlation between WA of TAD and RMC (–0.8 < *R* < –0.5, *p* < 0.05).

**TABLE 2:** Correlation and significance between WA value of brain region and KCs.

		LPFC	RPFC	LMC	RMC	LOC	ROC
TDD	R	–0.331	–0.188	–0.225	–0.272	0.299	0.445
	P	0.194	0.471	0.385	0.292	0.244	0.074
SVD	R	–0.327	–0.184	–0.221	–0.273	0.304	0.446
	P	0.201	0.480	0.395	0.289	0.236	0.073
TAD	R	–0.444	–0.288	–0.390	–0.500	0.112	0.325
	P	0.074	0.263	0.121	0.041*	0.669	0.203

Significant correlations are marked with *(p < 0.05).

## Discussion

Based on fNIRS measurements, this study mainly studied the effects of each task state on brain function. In the course of the experiment, it was observed that the activation and FC of the cortex of different brain regions were different in different task states. The main results are as follows:

(i) In grasping movements, the WA values of the LPFC, RPFC, LMC, RMC, and ROC shown upward trends compared with the resting state. The number and intensity of functional connections between channels increased during grasping movements compared with the resting state, but the changes were non-significant.
(ii) In VR grasping movements, the WA values of the LPFC, RPFC, LMC, RMC, and ROC were also increased compared with the resting state, and these differences were significant for the LPFC and LMC. The number of functional connections between the channels in each brain region decreased significantly during VR grasping movements compared with the resting state, but the WPCO value between the LPFC and LMC was significantly enhanced.
(iii) During VR grasping movements compared with grasping movements, the WA values of the LPFC, RPFC, RMC, and ROC were increased, and the WA of the LMC were increased significantly. Moreover, the number of functional connections between channels in each brain region decreased during VR grasping compared to non-VR grasping. In the LOC and LMC, the ROC and LMC, WPCO showed a significant decrease, on the contrary, the WPCO between the LPFC and LMC was improved.

Combining results (i) and (ii), we can infer that grasping tasks, with or without VR, can actively promote the activation of the cerebral cortex. However, in different task states, the changes in FC among brain regions followed quite the opposite pattern. During the VR grasping task, the number of functional connections to most cortical regions decreased. Some studies have shown that as the task difficulty increases, the brain areas that control unilateral movements will be more activated and will inhibit the FC of contralateral brain areas (Buetefisch et al., 2014). This phenomenon occurs because the degree of brain activation and the redistribution of brain resources are associated with neurovascular coupling control, and the distribution of brain resources can change the activation intensity of each brain region. The functional connectivity between brain regions also changes accordingly (Fishburn et al., 2014). Cerebral blood flow increased during the grasping exercise compared with the resting state, and the measured cerebral blood oxygen concentration naturally increased. However, the simple grasping action was not intense enough to elicit FC changes for most cortical areas. Although the LPFC, RPFC, LMC, RMC, and ROC were activated, their resources were not fully mobilized (Wickens, 2008), resulting in non-significant increases in the number of functional connections between brain regions, except for a significant increase between the LPFC and LMC, which controls the movements of the right hand. In contrast, VR grasping movements activated target brain regions more potently, which resulted in the reduced distribution of resources to other brain regions due to the limitation of total resources available to the brain. Therefore, the number and intensity of general functional connections within the brain were naturally reduced (Wickens, 2008). This mechanism maximizes the activity of the affected side of the brain, which is one of the goals of rehabilitation exercise.

Result (iii) indicates that the PFC, MC and OC were activated more strongly during grasping movements in a VR task than during simple repetitive grasping movements, especially in the LMC, which controls the movements of the right hand. On the other hand, it didn’t enhance the FC and WPCO between the OC with MC, OC with PFC after adding VR game task, and the exact opposite is the FC and WPCO had decreased significantly. At the same time, the FC and WPCO between the LMC with LPFC were significantly enhanced after adding VR game task. It seems to indicate that adding VR game tasks promotes the coupling between consciousness and motion control.

Table 2 shows an interesting result. The TDD, SVD, and TAD can characterize the stability of the control to some extent, and the increase of the TDD, SVD, and TAD indicates that the stability of the control becomes worse. Combining the results (ii), we can identify that there is a negative correlation between the WA of the LPFC, RPFC, LMC, RMC, and the TDD, SVD, and TAD of the VR grasping movements, and there is a significant negative correlation between the WA of RMC with the TAD, which seems to indicate that the more orderly the grasping action is, the stronger the activation of the LPFC, RPFC, LMC, and RMC is. At the same time, we observed that there was a positive correlation between the WA of LOC, ROC with the TDD, SVD, and TAD, while the LOC and ROC were visual feedback areas (Astafiev et al., 2004), which seems to indicate that increasing visual stimuli, such as pictures or videos, would lead to more unstable control during grasping movements. So when we add VR in the rehabilitation movements, we need to add more targeted images and remove irrelevant animation scenes.

In summary, grasping movements in a VR task can improve the activation and FC intensity of the ipsilateral brain region, inhibit the FC of the contralateral brain region, and reduce the allocation of brain resources to other brain regions. In the VR grasping exercises, ordered grasping movements can increase active participation, and promote the activation of the PFC and MC. But at the same time, excessive increase of the VR scene may reduce the training effect of grasping movements.

## Limitations

The purpose of using healthy subjects in this project was to test whether VR games can promote the rehabilitation efficiency of stroke patients and their enthusiasm to participate in training. This project also has the limitation of small sample size, and the following study will focus on the stroke patients. In the experimental design stage, we failed to disrupt the order of the project, but in fact disrupting the experimental sequence will be more beneficial to the results of this study. At the same time, in order to minimize the test variables, we didn’t add any other sounds in each experiment, but this also leads to the grasping movements and VR grasping movements can’t be at the same frequency, which needs to be improved in later projects.

## Data Availability Statement

The raw data supporting the conclusions of this article will be made available by the authors, without undue reservation.

## Ethics Statement

The studies involving human participants were reviewed and approved by the Human Ethics Committee of National Research Center for Rehabilitation Technical Aids. The patients/participants provided their written informed consent to participate in this study.

## Author Contributions

JL and ZL: conceptualization, investigation, supervision, project administration, and funding acquisition. XL, JL, JY, HL, and GX: methodology. XL, CH, HX, and WL: formal analysis. XL, JY, and HL: data curation. XL: writing—original draft preparation. XL and ZL: writing—review and editing. All authors have read and agreed to the published version of the manuscript.

## Conflict of Interest

The authors declare that the research was conducted in the absence of any commercial or financial relationships that could be construed as a potential conflict of interest.

## Publisher’s Note

All claims expressed in this article are solely those of the authors and do not necessarily represent those of their affiliated organizations, or those of the publisher, the editors and the reviewers. Any product that may be evaluated in this article, or claim that may be made by its manufacturer, is not guaranteed or endorsed by the publisher.

## Abbreviations

fNIRS: Functional near-infrared spectroscopy
PFC: Prefrontal cortex
MC: Motor cortex
OC: Occipital cortex
VR: Virtual reality
WA: Wavelet amplitude
WPCO: Wavelet phase coherence
TDD: Total distance difference
SVD: Sum velocity difference
TAD: Total acceleration difference
KCs: Kinematics characteristic
FC: Functional connectivity.
